# Control of HIV-1 Pathogenesis in Viremic Nonprogressors Is Independent of Gag-Specific Cytotoxic T Lymphocyte Responses

**DOI:** 10.1128/JVI.00346-18

**Published:** 2018-05-29

**Authors:** Maria Salgado, Albert Garcia-Minambres, Judith Dalmau, Esther Jiménez-Moyano, Pompeyo Viciana, Belén Alejos, Bonaventura Clotet, Julia G. Prado, Javier Martinez-Picado

**Affiliations:** a irsiCaixa AIDS Research Institute, Badalona, Spain; b Hospital Universitario Virgen del Rocio, Seville, Spain; c Centro Nacional de Epidemiología–ISCIII, Madrid, Spain; d University of Vic–Central University of Catalonia (UVic-UCC), Vic, Spain; e Catalan Institution for Research and Advanced Studies (ICREA), Barcelona, Spain; Emory University

**Keywords:** CTL response, HIV-1, viremic nonprogressors, progression, viral pathogenesis

## Abstract

Viremic nonprogressors (VNPs) constitute a very scarce group of untreated human immunodeficiency virus type 1 (HIV-1)-infected individuals who maintain stable CD4^+^ T cell counts despite high levels of HIV-1 replication. The specific factors associated with this atypical control of the HIV infection have been poorly described. Since specific T cell responses seem to be one of the main causes of HIV-1 control in elite controllers, we studied whether HIV-1 Gag-specific cytotoxic T lymphocyte (CTL) responses could also modulate disease control in VNPs. We characterized the immune responses from four VNPs compared to those of five standard progressors (SPs) during the first years of HIV-1 infection. We observed no differences in the breadth and frequency of Gag-specific cellular responses. Furthermore, we obtained 217 HIV-1^Gag^ clonal sequences in which the viral variability of Gag increased over 3 years of infection for synonymous and nonsynonymous mutations in both VNPs and SPs. VNPs evolution rates in *gag* were comparable to SPs. This observation is in line with a similar accumulation of CTL putative escape mutations in Gag epitopes targeted by CTL responses. Altogether, the absence of viral pathogenesis in VNP individuals seems to be independent of HIV-Gag-specific CTL responses. This novel information guides to the study of alternative mechanism of HIV-1 pathogenesis control.

**IMPORTANCE** Control of HIV infection has been widely studied in elite controllers or long-term nonprogressor models. However, there is a less-known group of individuals, termed viremic nonprogressors (VNPs), who maintain stable CD4^+^ T cell counts despite high plasma viremia. The mechanisms involved in this remarkable control of HIV-1 pathogenesis clearly have implications for the development of new drugs and vaccines. We show here for the first time that VNPs have immune responses and HIV-gag evolution similar to those of standard progressors. Remarkably, we demonstrate that the mechanism of pathogenesis control in these individuals differs from some elite controllers that are reported to have improved immune control. This is noteworthy since it opens the door to new, as-yet-unknown mechanisms for HIV control. Our novel results advance the understanding of mechanisms involved in viremic nonprogression and suggest that there are alternative mechanisms to the adaptive immune responses for an effective control of viral pathogenesis.

## INTRODUCTION

Untreated human immunodeficiency virus (HIV) infection leads to a progressive reduction on CD4^+^ T lymphocytes, which finally results in the development of AIDS-defining symptoms. This severe immunosuppression is typically associated with high levels of viral replication (1, 2). However, the termed HIV-1 viremic nonprogressors (VNPs) maintain stable CD4^+^ T cell counts despite high plasma viremia (3). This infrequent phenotype resembles the natural infection of sooty mangabeys and African green monkeys by the simian immunodeficiency virus (SIV) (4). SIV-infected natural host nonhuman primates rarely progress to AIDS and have normal life spans despite displaying high levels of viral replication. Both human VNPs and sooty mangabeys have the common characteristic of presenting low immune activation despite the high viremia (3, 5).

The specific viral and host factors associated with this atypical control of the HIV-1 infection are poorly described. Recent data suggest that VNPs might be infected with virus with impaired fitness (6), although previous studies showed no significant alterations in *env*- and *nef*-associated functions (7, 8). On the other side, limited infection of Tcm and Tscm CD4^+^ T cells in VNPs has been reported (9), suggesting that host factors rather than specific viral properties may allow VNPs to dodge HIV-1 pathogenesis and chronic immune activation. Research performed in nonhuman primates have suggested that SIV-specific cytotoxic T lymphocyte (CTL)-mediated immune responses are not responsible for the lack of disease progression in the SIV natural host (10, 11). However, no further immunological studies have been performed in humans.

An effective HIV-specific CTL response is postulated as one of the main causes of HIV-1 control in elite controllers (12), but there are no studies addressing whether VNPs could avoid the HIV-1 disease progression through alternative CTL-related mechanisms. It is therefore essential to understand whether cellular HIV-specific immune responses play a role in VNPs. This study evaluates whether HIV-specific CTL responses are effective in controlling the viral pathogenesis in VNPs compared to HIV-infected standard progressors (SPs) during 3 years in the absence of combination antiretroviral therapy. Adaptive immune pressure in a highly replicating viral environment might contribute to select viral mutants with impaired viral fitness and consequently reduced viral pathogenesis.

## RESULTS

### Subjects.

Clinical characteristics from the studied individuals are depicted in Table 1. VNPs and SPs presented similar plasma viremia (4.6 ± 0.22 log HIV RNA copies/ml versus 4.4 ± 0.23 log HIV RNA copies/ml, respectively). The SP group had significantly lower CD4^+^ T cell levels (VNP [707 ± 215 cells/μl] versus SP [216 ± 308 cells/μl]), with greater cell decay over time (VNP [23 ± 60 cells/μl/yr] versus SP [117 ± 6 cells/μl/yr]). The time from the seroconversion year of the first sample (*t*_0_) was similar in both groups.

**TABLE 1:** Clinical data^*a*^

Group	Patient ID	Age (yr)	Gender	Estimated seroconversion date (yr)	Yr sampled (*t*_0_)	Period (yrs) between *t*_0_ and *t*_3_ samples	CD4 decay (cells/μl/yr)	CD4 count (cells/μl) at *t*_3_	Mean VL (log copies HIV-RNA/ml)	HIV subtype	HLA allele(s)
VNP	VNP-1	33	Male	2007	2008	3.3	−74	632	5.0	B	A*0301, A*3201, B*4403, B*4901, Cw*0701, Cw*1601
	VNP-2	30	Male	2008	2009	3.1	−56	999	4.3	B	A*0201, A*3201, B*4402, B*4402, Cw*0501, Cw*0501
	VNP-3	28	Male	2006	2008	2.7	16	593	4.7	B	A*0201, A*0201, B*0702, B*5101, Cw*0702, CW*1502
	VNP-4	31	Male	2006	2008	2.6	10	783	4.6	CRF19	A*2902, A*6802, B*4403, B*5301, Cw*0401, Cw*1601
SP	SP-1	40	Male	2000	2001	3.1	−115	468	4.4	B	A*0201, A*6802, B*1402, B*1801, Cw*0501, Cw*0802
	SP-2	47	Male	2007	2008	3.2	−117	342	4.2	B	A*1101, A*3001, B*1302, B*1501, Cw*0303, Cw*0602
	SP-3	43	Male	2005	2007	2.6	−121	34	4.1	B	A*2402, A*2601, B*4405, B*5101, Cw*0202, Cw*1402
	SP-4	38	Male	2006	2007	2.1	−234	352	5.0	B	A*0301, A*3002, B*1801, B*3503, Cw*04, Cw*05
	SP-5	44	Male	2007	2008	2.3	−110	216	4.5	B	A*0301, A*2902, B*4001, B*4403, Cw*0304, Cw*1601

a For the epidemiological data, the age, estimated seroconversion date, time between samples, CD4 counts at time point *t*_3_, CD4 decays, mean viral load, HIV-1 subtype, and HLAs are detailed for each individual. The VNP and SP groups showed differences in CD4 counts and CD4 decays but not in mean viral loads.

### CTL responses between VNPs and SPs.

HLA class I was genotyped for all the individuals (Table 1). None of the participants carried protective HLA allele B*5701 or B*2701. Rapid progression alleles (B*3503) were also absent in both groups.

In order to characterize HIV-specific CTL responses in the VNP group, we assessed the production of gamma interferon (IFN-γ) by peripheral blood mononuclear cells (PBMCs) stimulated with Gag peptides. Longitudinal analysis was only performed in the VNPs, since PBMC samples were not available for SPs at *t*_0_. Nonetheless, comparisons between VNPs and SPs were performed at *t*_3_.

**FIG 1:**
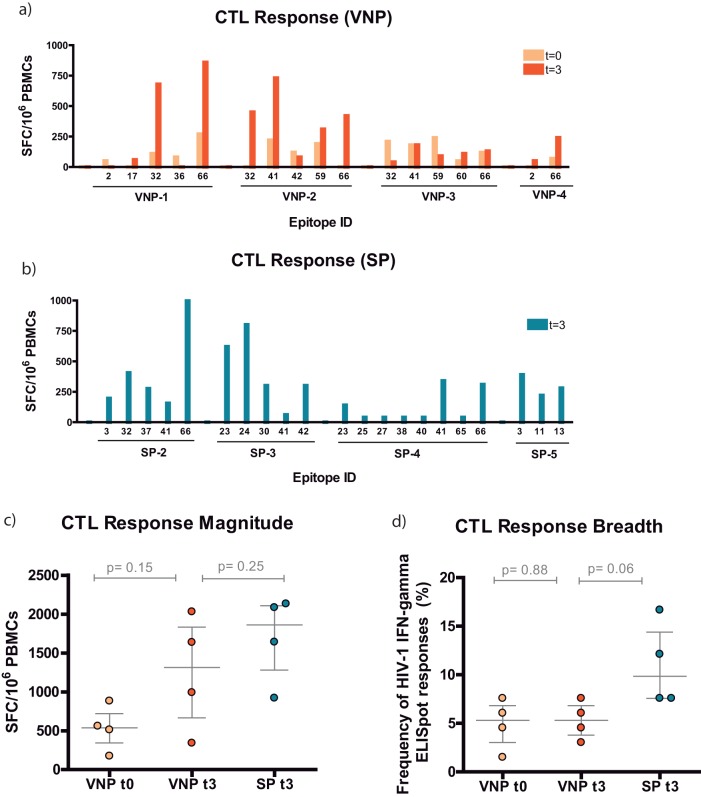
Epitope-specific CTL response in VNPs and SPs. (a and b) Map of the specific overlapping epitopes that generated IFN-γ responses for VNPs and SPs, respectively. Data are presented as spot-forming cells (SFC) per 10^6^ PBMCs. Specifically, for VNPs we were able to compare samples from *t*_0_ (light orange) with those from *t*_3_ (dark orange). (c) Specific CTL response magnitude. The magnitude of the response was the additive response of all the positive wells measured as SFC/10^6^ PBMCs. VNP data are represented in light orange (t_0_) or dark orange (t_3_). SP data are presented in dark blue (t_3_). (d) Specific CTL response breadth.

CTL responses per subject are depicted in Fig. 1a and b. Specific epitopes in p24^Gag^ and p6^Gag^—32 (MREPRGSDIAGTTSTL), 41 (YVDRFYKTLRAEQASQEV), and 66 (KELYPLASLRSLFGNDPSSQ)—were overrepresented in both VNPs and SPs. Those regions seemed to be immunodominant in the early phase of the HIV-1 infection. The magnitudes of the responses slightly increased over time in VNPs, but not their breadths (Fig. 1c and d). When both groups were compared at *t*_3_, similar responses were found, but the frequency was slightly higher in SPs (Fig. 1c and d). Together, these data suggest that VNPs and SPs presented similar T cell HIV-specific responses, although the breadth tends to be wider in SPs.

### Viral evolution in VNPs and SPs.

**FIG 2:**
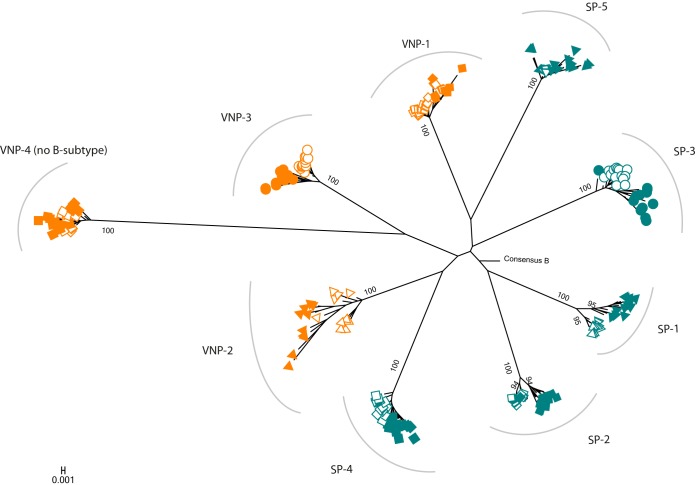
Phylogenetic tree of *gag* clonal sequences from VNPs and SPs. VNP sequences are indicated in orange, and SP sequences are indicated in blue. Sequences from *t*_0_ are represented as open symbols and from *t*_3_ as filled symbols.

In order to identify the impact of HIV-specific CTL responses in viral evolution, we analyzed 217 clonal Gag sequences obtained from plasma by limiting dilution. For each individual, we obtained a median of 12 independent Gag sequences (range, 10 to 14) in two different time points separated by 2.5 to 3 years. In all cases, sequences grouped independently per subject (Fig. 2). Since VNP-4 carried non-B subtype virus, these sequences clustered distantly from the rest. Gag sequences diverged over time in all subjects, with *t*_0_ and *t*_3_ clustering separately in most cases.

**FIG 3:**
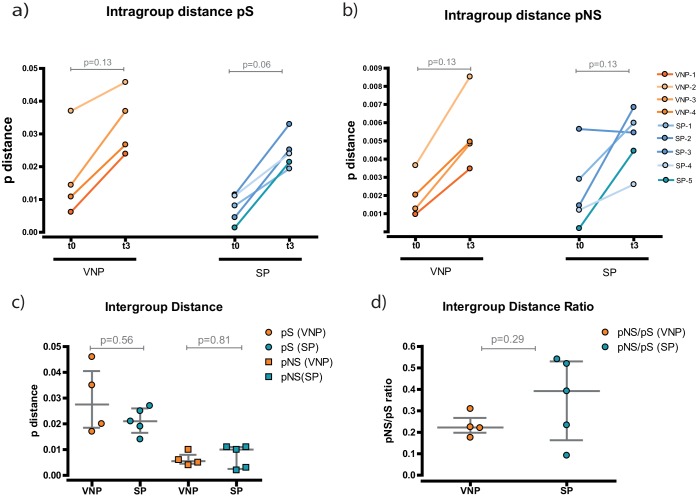
Evolution analysis of the gag sequences. Diversity is measured as the p-distance measuring the proportion of changes in each sequence calculated as nucleotide (synonymous) or amino acid changes (nonsynonymous). (a and b) Intragroup distances. Variability in each subject's group of sequences was determined at each time point. Each participant is represented by a different shade of orange (VNPs) or blue (SPs). (c) Intergroup distances. Evolution over time was determined for each participant. VNP samples are indicated by orange symbols and SP samples by blue. Synonymous evolution is indicated by circles and nonsynonymous evolution by squares. (d) Intergroup distance ratio. pNS/pS ratios were determined. VNP samples are indicated in orange and SP samples in blue.

Using a p-distance algorithm, we calculated the diversity of the sequences represented in the tree. This parameter measures the proportion of changes in each sequence calculated as synonymous (pS) or nonsynonymous (pNS) (Fig. 3). Consistently, we observed a longitudinal increase in genetic diversity over time for both VNPs and SPs (Fig. 3a and b), although statistically significant differences were not reached. This supports that genetic diversity increases over time, as suggested by the phylogenetic tree.

To compare whether the degree of diversity is different between VNPs and SPs, we measured the global evolution per individual (Fig. 3c). We observed no changes between the two groups in either synonymous or nonsynonymous mutations. Likewise, there were no differences when we analyzed Gag regions p7, p24, or p2 separately (data not shown). Finally, we checked whether there was positive evolution by examining the pNS/pS ratio. No significant differences were found, and both ratios were <1, indicating that there was not a predominant positive selection in any of the groups (Fig. 3d).

Altogether, these data reflect continuous viral evolution between VNP and SP during the study period despite differences in viral pathogenesis. Therefore, no changes in the rate of evolution were observed between groups.

### CTL-associated epitope evolution.

**FIG 4:**
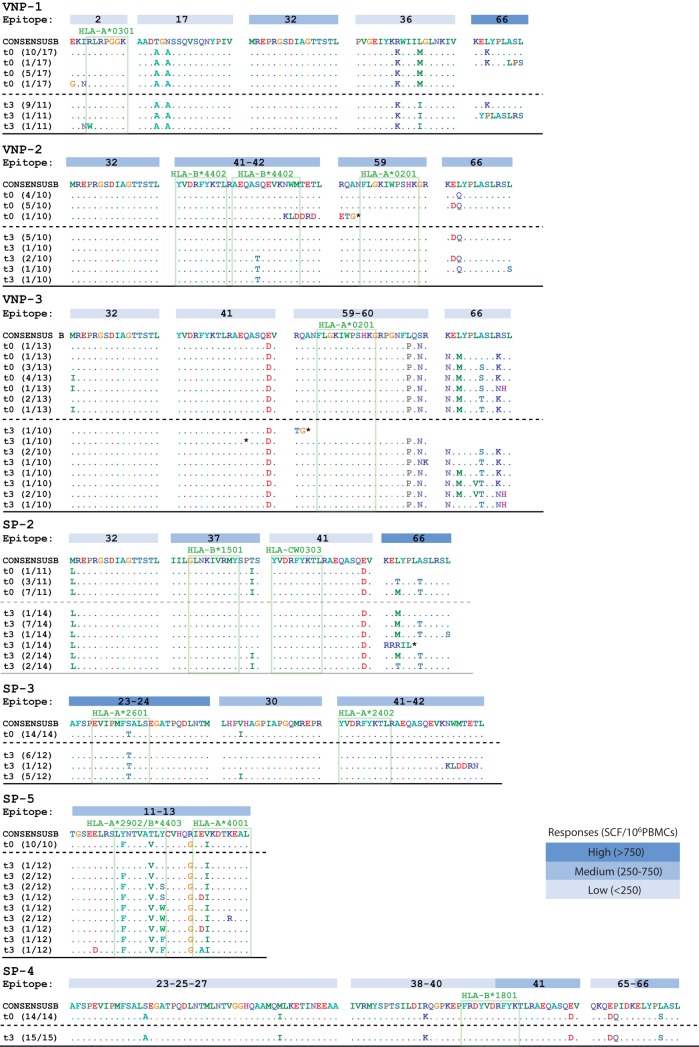
Genetic variation in epitopes with positive responses in each subject. Blue boxes represents the relative intensity IFN-γ responses in each epitope. VNP-4 responses are not shown since they are outside the sequenced area. The SP-9 immune response was not determined due to the bad cell quality. Open boxes represents HLA-associated epitopes.

We further evaluated the appearance of Gag immune mutants as an indirect measure of CTL pressure. For each individual, we checked intraepitope viral evolution during follow-up (Fig. 4). Both groups developed putative escape mutations in gag epitopes during follow-up. The mutations were mainly concentrated in the regions shared with a specific HLA-restricted epitope, but also potential compensatory adjacent mutations were observed. Putative escape mutation appeared in epitopes that induced low-, medium-, and high-magnitude CTL responses, as shown in Fig. 4.

## DISCUSSION

Due to the rarity of the VNP phenotype among HIV-infected individuals, few cohorts with accurate follow-up have been described. In this study, we monitored VNPs and SPs longitudinally in order to understand whether cellular HIV-specific immune responses play a role in controlling viral pathogenesis. We observed no improved immune response in the VNP group, as happens in other HIV-1 nonprogression phenotypes such as elite controllers or long-term nonprogressors (13, 14).

We observed how *gag* evolution increased over time similarly in VNPs and SPs. Differences were not observed when we studied nucleotides or amino acid changes. However, when we analyzed the variation in the epitopes with positive responses, we observed that these areas were more variable at the study baseline in VNPs than in SPs. The number of Gag CTL escape mutants has been previously associated with viral replicative capacity (15). Since putative escape mutations happen later at the same level in both groups, it seems that this is not conferring an advantage to the VNPs to sidestep viral pathogenesis. This contrasts with observations in other cohorts with nonpathogenic infection as the elite controllers. Previous work performed in elite controllers associated a strong immune response against Gag epitopes with a sustained control of the HIV-1 disease (16) and epitope-dependent evolution. In the present study, we chose the *gag* gene because it is the most conserved gene in the entire HIV-1 genome, and the majority of CTL immunodominant responses are directed against Gag. However, additional analyses of HIV-1 Env evolution might provide additional information linked to differential humoral response between groups, as previously described (8).

Major distinguishing features of SIV infection in its natural hosts (African green monkeys and sooty mangabeys) include the absence of disease progression (despite displaying high viremia and high viral replication rates in the intestine), the rapid resolution of virus-induced inflammation, the lack of microbial translocation, rapid control of viral replication in secondary lymphoid organs, and a lack of viral trapping by follicular dendritic cells (FDCs) in follicles (17–24). Recent data demonstrate that, in nonpathogenic SIV infection of African green monkeys, natural killer cells accumulate in an interleukin-15-dependent manner in the follicles of secondary lymphoid organs of the animals and exert efficient control of viral replication within lymph nodes (25). Therefore, the role of NK cells should also be explored in HIV-infected subjects with a VNP phenotype to understand their contribution in limiting viral pathogenesis. However, the extremely scarce number of HIV-infected individuals with this nonprogressive disease profile, along with the current recommendations of rapid antiretroviral therapy initiation in all patients irrespective of the time since HIV-1 transmission (26), may limit the investigation of this hypothesis. In this sense, the conclusions of our study are conditioned to its small sample size. Likewise, the absence of baseline samples for SPs limited our power to reach robust conclusions when global immune responses were measured. However, this limitation was compensated for by performing an extensive study of the immunological marks within the viral genome using a significant number of clonal sequences per individual.

To our knowledge, this is the first report evaluating the immune cytotoxic response in VNP HIV-infected individuals. We here demonstrated that HIV-specific CTL responses are not responsible for the control of HIV-induced pathogenesis in subjects with an absence of CD4 T-cell depletion despite high viral replication. Alternative mechanisms, including humoral responses or differential infection of T cell CD4^+^ subsets, might be an alternative mechanism of protection against HIV-mediated immune depletion. Further research will unravel whether the VNP phenotype is associated with additional host factors beyond the scope of the present study.

## MATERIALS AND METHODS

### Subjects.

We selected four VNPs and five untreated HIV-infected SPs from the IrsiCaixa VNPs Cohort and the Spanish AIDS Research Network Cohort (CoRIS) (27). VNPs were defined as HIV-infected subjects with viral loads above 10,000 HIV RNA copies/ml that maintained CD4^+^ T cells counts above 500 cells/μl for more than 5 years with decays of less than 100 cells/μl/year. SPs are HIV-infected subjects within the same range of plasma viremia but with a decay in CD4^+^ T cells counts greater than 100 cells/μl/year, falling below 500 cells/μl after 3 years of monitoring. All of the samples were retrospectively obtained from naive individuals within 4 years after infection. The seroconversion date was estimated using last negative HIV test.

### HLA typing and assessment of HIV-specific CTL responses.

High-resolution HLA class I typing for alleles A, B, and Cw was performed at the Blood and Tissue Bank of Barcelona. Comprehensive HIV-1 epitope screening of optimal responses was carried out using the IFN-γ ELISpot assay, as previously described (28). PBMCs were stimulated with a bulk of 66 clade B consensus overlapping peptides covering the entire Gag protein. Wells were considered positive above the background level, as previously reported (29). The breadth of HIV-specific responses was calculated as follows: (number of positive responses/number of peptides tested) × 100. The magnitude of response was the additive response of all the positive wells.

### Gag amplification and phylogenetic analyses.

Plasma samples were collected at two time points per individual separated by 2 to 3 years (*t*_0_ and *t*_3_). Viral RNA was isolated from plasma (Qiagen) and retrotranscribed in cDNA using the 3′-outer primer and SSIII retrotranscriptase (Invitrogen). To prevent resampling, *gag* was amplified from cDNA using limiting-dilution clonal nested PCR as previously described (30). To ensure single gene amplification, we only sequenced positive PCRs if less of 25% of the reactions were positive. This translated to a minimum of 86% probability of clonality according to Poisson distribution. Additional quality control was done by discarding residual sequences with double peaks in the chromatograms. Only *gag* clonal sequences were included in the phylogenetic analysis. Sequences were assembled using Sequencher 5.0, and the alignments were manually adjusted in Bioedit. Maximum-likelihood analyses with a Kimura two-parameter phylogenetic reconstruction were performed. Nonsynonymous and synonymous p-distances were calculated on the Nei-Gojobori algorithm by comparing grouped sequences from *t*_0_ and *t*_3_ for each subject using MEGA 4.0 software (31).

### Statistical analyses.

Statistical analysis was performed using a Wilcoxon paired test or a Mann-Whitney test for paired or unpaired data, respectively.
